# International Spread of Multidrug-Resistant *Rhodococcus equi*

**DOI:** 10.3201/eid2809.220222

**Published:** 2022-09

**Authors:** Jorge Val-Calvo, Jane Darcy, James Gibbons, Alan Creighton, Claire Egan, Thomas Buckley, Achim Schmalenberger, Ursula Fogarty, Mariela Scortti, José A. Vázquez-Boland

**Affiliations:** University of Edinburgh, Edinburgh, Scotland, UK (J. Val-Calvo, M. Scortti, J.A. Vazquez-Boland);; University of Limerick, Limerick, Ireland (J. Darcy, A. Schmalenberger);; Irish Equine Centre, Naas, Ireland (J. Gibbons, A. Creighton, C. Egan, T. Buckley, U. Fogarty)

**Keywords:** *Rhodococcus equi*, multidrug-resistant *Rhodococcus equi*, MDR *R. equi*, MDR-RE, *R. equi* MDR 2287 clone, *erm*(46), pRErm46, Tn*RErm46*, macrolide resistance, rifampin resistance, bacteria, antimicrobial resistance, zoonoses, United States

## Abstract

A multidrug-resistant clone of the animal and human pathogen *Rhodococcus equi*, MDR-RE 2287, has been circulating among equine farms in the United States since the 2000s. We report the detection of MDR-RE 2287 outside the United States. Our finding highlights the risk for MDR-RE spreading internationally with horse movements.

*Rhodococcus equi* is a soilborne aerobic actinomycete bacterium that infects animals and humans. Human infections are opportunistic, zoonotic in origin, and considered to be linked to exposure to farm environments (*1*–*3*). Although clinical *R. equi* infections are relatively rare in most animal species, foals are commonly affected, and incidence is often high in horse farms in equine breeding countries (*4*). Long courses of rifampin and a macrolide is the mainstay therapy for foal rhodococcosis. This treatment has been systematically used since the 1980s, and no significant resistance was detected until the early 2000s, after mass prophylactic application at *R. equi*–endemic farms in the United States (*5*,*6*). 

The emerging dual macrolide–rifampin resistance is attributable to a multidrug-resistant *R. equi* (MDR-RE) clone, named 2287, which has spread among horse farms across the United States. MDR-RE 2287 arose by co-acquisition of the conjugative plasmid pRErm46 and a specific *rpoB*^S531F^ (TCG to TTC) mutation conferring high-level rifampin resistance (*7*,*8*). pRErm46 specifies resistance to macrolides, lincosamides, and streptogramins via the *erm*(46) gene carried on Tn*RErm46,* a highly mobile transposon, and to sulfonamides, streptomycin, spectinomycin, tetracycline, and doxycycline via a class 1 integron (C1I) and associated *tetRA*(33) determinant (*9*). Although it has so far been found only in the United States, we predicted MDR-RE could spread to other countries with the movement of equids (*10*).

## The Study

After MDR-RE was characterized in 2019 (*8*), we established an informal surveillance network with colleagues in North and South America, Europe, the United Kingdom, Africa, Asia, and Australia. We asked collaborating laboratories to review their retrospective *R. equi* collections and prospectively identify isolates with erythromycin MIC >4 μg/mL, potentially denoting *erm*(46)-mediated macrolide resistance. Two equine clinical strains from necropsied foals in Ireland met the criterion: PAM2528, recovered in 2016, and PAM2578, recovered in 2021 (henceforth designated as 2528 and 2578). Both strains originated from the same farm and had MICs >32 μg/mL for erythromycin and >256 μg/mL for rifampin, consistent with MDR-RE’s resistance phenotype (*7*–*10*).

**Figure 1:**
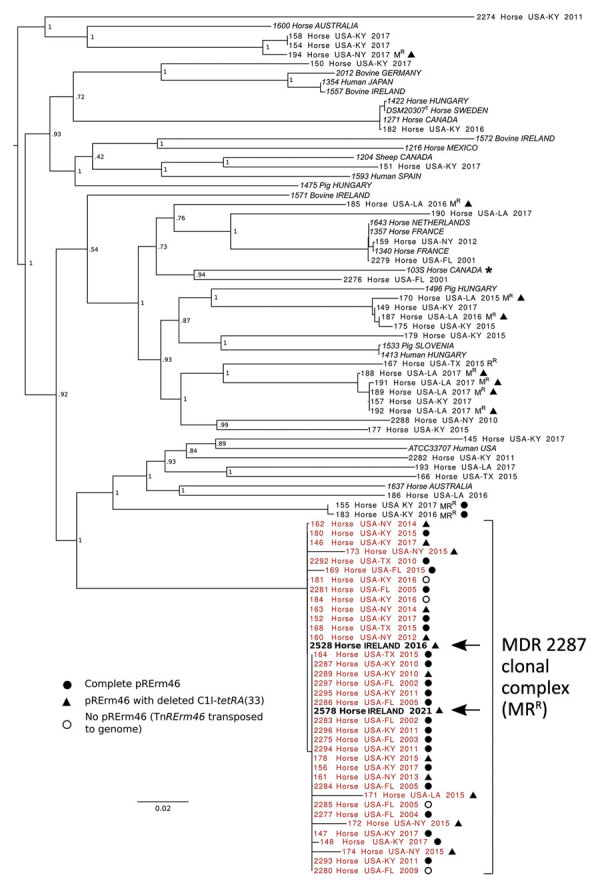
Whole-genome phylogenetic analysis of *Rhodococcus equi* and its multidrug-resistant 2287 clone. Asterisk indicates strain 103S used as reference genome (GenBank accession no. FN563149). For analysis we used 92 *R. equi* genome sequences including 68 macrolide-resistant and -susceptible equine isolates from the United States and 22 global strains from a previously reported *R. equi* diversity set (*14*) (italics). Macrolide-resistant isolates include 36 members of the MDR-RE 2287 clonal complex (red text) as well as isolates representing spillages of the pRErm46 plasmid to other *R. equi* genotypes (*8*,*10*). Arrows indicate the 2 MDR-RE 2287 isolates from Ireland. Labels indicate geographic origin, year of isolation, and resistance phenotype when applicable (MR^R^, macrolide and rifampin resistance; M^R^, macrolide resistance; R^R^, rifampin resistance). Symbols indicate pRErm46 carriage in macrolide-resistant isolates, described in the key; open circles indicate MDR-RE isolates where pRErm46 has been lost after transposition of the Tn*RErm46* element to the host genome (*8*). Numbers at nodes indicate bootstrap values for 1,000 replicates. Tree was drawn with FigTree (http://tree.bio.ed.ac.uk/software/figtree).

We confirmed that both isolates were *erm*(46) positive by PCR and carried the *rpoB*^S531F^ mutation unique to the MDR-RE 2287 clone using previously described methods (*9*). A PCR using primers 5′-CCGAGATGTGTCGGACTTC-3′ (forward) and 5′-CGCCGAAGAACAACCCGAGGATG-3′ (reverse) showed the pRErm46 resistance plasmid carried the ΔC1I-*tetRA* deletion, observed in some recent MDR-RE isolates (*9*,*10*). Accordingly, 2528 and 2578 were susceptible to trimethoprim/sulfamethoxazole, streptomycin, spectinomycin, and tetracycline, to which the C1I-*tetRA*(33) determinant confers resistance (*9*). We used paired-end Illumina sequencing (Illumina, https://www.illumina.com) to obtain genomic libraries of the isolates. Reads were quality-checked using FastQC version 0.11.9 (https://www.bioinformatics.babraham.ac.uk/projects/fastqc), trimmed with TrimmomaticPE version 0.39 (*11*), and assembled using SPAdes v3.15.2 (*12*). For 2528 sequences, average forward/reverse Phred score was 38/38, coverage depth 79×, and number of contigs (>1 kb) 117. For 2578, average Phred score was 36/36; coverage depth, 243×; and number of contigs, 26. BlastN searches (https://blast.ncbi.nlm.nih.gov/Blast.cgi) confirmed presence of pRErm46 sequences in the draft genomes. We used ParSNP version 1.5.6 (*13*) and FastTree version 2.1.11 (http://www.microbesonline.org/fasttree) to build approximate maximum-likelihood trees based on core single-nucleotide polymorphisms (SNPs) to determine the position of the isolates in the *R. equi* population structure. The output *R. equi* tree showed that the 2 resistant isolates from Ireland belonged to the MDR-RE 2287 clone (Figure 1).

**Figure 2:**
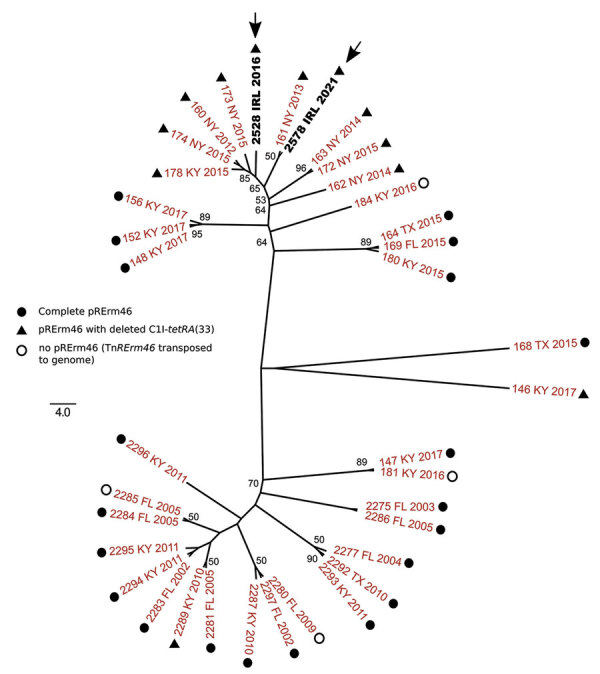
Unrooted maximum-likelihood tree of multidrug-resistant *Rhodococcus equi* 2287 clonal complex showing the relationships of the isolates from Ireland (arrows). Whole-genome phylogeny inferred from 45 parsimony informative sites using SNIPPY (https://github.com/tseemann/snippy) and IQ-tree (http://www.iqtree.org) for tree reconstruction. Best-fit model was selected by IQ-tree’s ModelFinder module. Bootstrap values >50 are shown. The genome of the prototype MDR-RE 2287 isolate PAM2287 (National Center for Biotechnology Information assembly accession no. GCA_002094405.1) was used as a reference for SNP calling. Labels indicate isolate name, geographic origin (FL, Florida; IRL, Ireland, KY, Kentucky; NY, New York; TX, Texas), and year of isolation. Symbols indicate pRErm46 plasmid type. Tree was drawn with FigTree (http://tree.bio.ed.ac.uk/software/figtree).

Because the short genetic distances compressed the branching of the MDR-RE 2287 isolates, we repeated the phylogenetic analysis with only the clonal genomes to explore their relationships in more detail (Figure 2). For this analysis, we detected core SNPs using SNIPPY version 4.6.0 (https://github.com/tseemann/snippy), which avoided genome alignment errors observed with ParSNP that distort the phylogenetic reconstruction of virtually identical isolates.

The consensus maximum-likelihood tree subdivided the MDR-RE 2287 clonal complex in 2 main sublineages, one comprising older isolates from 2002–2011 and the other isolates from 2015 and later. *R. equi* 2528 and 2578 were located in 2 adjacent top branches within the younger sublineage, together with all (n = 7) isolates from New York. Because the analyzed MDR-RE 2287 collection comprised isolates from different US locations, the clustering with the New York isolates suggested a common origin. The New York isolates were recovered over a period of several years since 2012, pointing to a locally circulating MDR-RE 2287 subpopulation as the likely source of the isolates later found in Ireland. This possibility was further supported by the finding that both the 2528 and 2578 genomes possessed ΔC1I-*tetRA* pRErm46 variants, also carried by the New York subpopulation but only exceptionally by other MDR-RE 2287 complex members (Figures 1, 2).

## Conclusions

We document the international spread of the MDR-RE 2287 clone that has been circulating in the United States since the 2000s (*8*,*10*). MDR-RE 2287 appears to be following the same pattern of the pandemic multidrug-resistant clones of human bacterial pathogens, which within a few years after emergence and initial local expansion become globally disseminated (*15*). For MDR-RE, the process is slower, likely because of fewer opportunities for transmission afforded by horse trade and contacts compared with the scale of human interactions and travel.

The positioning of the Ireland isolates in 2 separate subbranches of the New York radiation (Figure 2) may indicate they represent independent, temporally distinct import events that took place around 2016 and 2021, involving different MDR-RE 2287 subclones. That MDR-RE 2287 was not detected again in Ireland until 5 years later indicates that it might not have persisted after its first appearance in the country in 2016. This scenario may be explained by the different *R. equi*–targeted equine farm management in Ireland compared with that in the United States, where the emergence, maintenance, and spread of MDR-RE was favored by the application of mass antibioprophylaxis at *R. equi*–endemic farms (*6*,*8*). This practice was not implemented on the affected farm, nor is it applied in Ireland in general. However, the genetic distance between the 2528 and 2578 strains is comparable to that for the 7 New York isolates, recovered during 2012–2015. It cannot therefore be excluded that the 2 Ireland strains represent successive isolations of a locally evolving single imported MDR-RE 2287 subclone (21 SNPs difference over 5M bp, ≈1 × 10^−6^ substitutions/site/year, consistent with normal genetic drift values).

A Kentucky isolate of the ΔC1I-*tetRA* type was also located in a terminal branch of the New York cluster, whereas Kentucky isolates with complete pRErm46 plasmids were positioned at basal bifurcations of the radiation (e.g., the 148, 152, and 153 clusters) (Figure 2). These observations suggest a transmission history in which a relatively recent MDR-RE 2287 subclone that acquired a pRErm46 ΔC1I-*tetRA* deletion, possibly originating from Kentucky where MDR-RE emerged and is prevalent (*8*,*9*), became endemic in a New York farm and was transferred, directly or indirectly, to Ireland. International trade in thoroughbred horses is frequent, and the affected farm in Ireland received horses from the United States, Europe, and the United Kingdom on a regular basis. Previous phylogenomic studies provided evidence of global circulation of *R. equi* genomotypes, probably linked to livestock trade (*14*). Our findings reinforce this notion and warn about the risk for MDR-RE becoming globally disseminated over time with horse movements.

Our study is not comprehensive but based on the voluntary collaboration of a small number of international colleagues, and thus MDR-RE may have also spread to other countries. We recommend actively monitoring the occurrence of the emerging MDR-RE 2287 clone for which, as our data highlight, *erm*(46) and the *rpoB*^S531F^ (TCG→TTC) mutation can be used as molecular markers, eventually complemented with pRErm46/ΔC1I-*tetRA*(33) variant detection.
